# Biofilm Formation and Motility Are Promoted by Cj0588-Directed Methylation of rRNA in *Campylobacter jejuni*

**DOI:** 10.3389/fcimb.2017.00533

**Published:** 2018-01-18

**Authors:** Agnieszka Sałamaszyńska-Guz, Simon Rose, Claus A. Lykkebo, Bartłomiej Taciak, Paweł Bącal, Tomasz Uśpieński, Stephen Douthwaite

**Affiliations:** ^1^Division of Microbiology, Department of Pre-Clinical Sciences, Faculty of Veterinary Medicine, Warsaw University of Life Sciences, Warsaw, Poland; ^2^Department of Biochemistry and Molecular Biology, University of Southern Denmark, Odense, Denmark; ^3^Division of Physiology, Department of Physiological Sciences, Faculty of Veterinary Medicine, Warsaw University of Life Sciences, Warsaw, Poland; ^4^Laboratory of Theory and Applications of Electrodes, Faculty of Chemistry, University of Warsaw, Warsaw, Poland

**Keywords:** TlyA 2′-*O*-methyltransferase, biofilms, bacterial motility, virulence, capreomycin resistance

## Abstract

Numerous bacterial pathogens express an ortholog of the enzyme TlyA, which is an rRNA 2′-*O*-methyltransferase associated with resistance to cyclic peptide antibiotics such as capreomycin. Several other virulence traits have also been attributed to TlyA, and these appear to be unrelated to its methyltransferase activity. The bacterial pathogen *Campylobacter jejuni* possesses the TlyA homolog Cj0588, which has been shown to contribute to virulence. Here, we investigate the mechanism of Cj0588 action and demonstrate that it is a type I homolog of TlyA that 2′-*O*-methylates 23S rRNA nucleotide C1920. This same specific function is retained by Cj0588 both *in vitro* and also when expressed in *Escherichia coli*. Deletion of the *cj0588* gene in *C. jejuni* or substitution with alanine of K^80^, D^162^, or K^188^ in the catalytic center of the enzyme cause complete loss of 2′-*O*-methylation activity. Cofactor interactions remain unchanged and binding affinity to the ribosomal substrate is only slightly reduced, indicating that the inactivated proteins are folded correctly. The substitution mutations thus dissociate the 2′-*O*-methylation function of Cj0588/TlyA from any other putative roles that the protein might play. *C. jejuni* strains expressing catalytically inactive versions of Cj0588 have the same phenotype as *cj0588*-null mutants, and show altered tolerance to capreomycin due to perturbed ribosomal subunit association, reduced motility and impaired ability to form biofilms. These functions are reestablished when methyltransferase activity is restored and we conclude that the contribution of Cj0588 to virulence in *C. jejuni* is a consequence of the enzyme's ability to methylate its rRNA.

## Introduction

Proteins encoded by orthologs of *tlyA* genes are present in a diverse range of bacterial pathogens including *Mycobacterium* sp., *Campylobacter jejuni* and *Brachyspira (Serpulina) hyodysenteriae*, and have been linked to various roles in virulence including hemolysis (Wren et al., 1998) and antibiotic resistance (Maus et al., 2005). The role of TlyA in resistance to the clinically important tuberactinomycin antibiotics capreomycin and viomycin is associated with its function as an rRNA methyltransferase (Johansen et al., 2006). TlyA 2′-*O*-methylates specific cytidines within the ribosomal binding site of capreomycin and viomycin. Inactivation of TlyA, with consequent loss of methylation, confers resistance in bacterial pathogens including *Mycobacterium tuberculosis* (Maus et al., 2005; Johansen et al., 2006; Monshupanee et al., 2012). Less clear, however, is how TlyA might engage in other aspects of virulence such as hemolysis that have been ascribed to this protein (Wren et al., 1998; Monshupanee, 2013).

Alignments of TlyA sequences from various organisms show that they possess a conserved K-D-K-E amino acid tetrad previously identified at the active core of other 2′-*O*-methyltransferases such as RrmJ/FtsJ (Bugl et al., 2000; Hager et al., 2002; Feder et al., 2003; Punekar et al., 2012). These structural features are also evident in the TlyA ortholog Cj0588 of *C. jejuni*. We show here that Cj0588 belongs to the type I group of TlyA (TlyA^I^) enzymes, which 2′-*O*-methylate solely at 23S rRNA nucleotide C1920 as previously seen for the *B. hyodysenteriae* and *Thermus thermophilus* homologs (Monshupanee et al., 2012). Slightly longer TlyA^II^ versions with sequence extensions at their N- and C-termini are found in actinobacterial species including *M. tuberculosis*, and these enzymes 2′-*O*-methylate not only nucleotide C1920 but also nucleotide C1409 in 16S rRNA (Johansen et al., 2006; Monshupanee et al., 2012). Nucleotides C1409 and C1920 are respectively located on the interface of the 30 and 50S ribosomal subunits and come into close proximity at the capreomycin/viomycin binding site upon subunit association (Johansen et al., 2006; Stanley et al., 2010).

In this study, we engineered Cj0588/TlyA in pathogenic strains of *C. jejuni* to produce experimental model systems for assessing the role of the protein in physiological traits such as motility and biofilm formation that have been linked with virulence (Young et al., 2007; Bolton, 2015). Biofilms are resilient structures that account for the prevalence of *C. jejuni* in food processing environments (Reuter et al., 2010), and are directly involved in human disease by facilitating adhesion of *C. jejuni* and its colonization of the mucus lining of the intestinal tract (Haddock et al., 2010). Adherence of *C. jejuni* to human colorectal epithelial cells has previously been shown to be impaired after loss of *cj0588* (Salamaszynska-Guz and Klimuszko, 2008), although it remained unclear how *cj0588* might function in this manner.

The motility of *C. jejuni* and its ability to form biofilms in the environment and during host infection are processes that can be reproduced and studied under laboratory conditions. Many commonly known *C. jejuni* strains that are pathogenic in humans (Epps et al., 2013) have been isolated from cases of zoonotic infection acquired from poultry and other animals (Luethy et al., 2017). *C. jejuni* 405 and 81-176 (Turonova et al., 2015) are representative strains with the former being the more motile and the latter more proficient at forming biofilms. We have engineered both strains with a series of amino acid substitution within the active site of the Cj0588 enzyme to cause loss of its TlyA^I^ methyltransferase activity without affecting the overall structure of the protein. Methylation of the *C. jejuni* rRNA was followed by biochemical and mass spectrometric approaches and was correlated with changes in phenotypic traits including motility and biofilm morphology.

## Materials and methods

### Modeling of the Cj0588/TlyA methyltransferase structure

The *C. jejuni* TlyA homolog Cj0588 (A0A0M4TPK5) was identified in BLAST searches (Altschul et al., 1997) using the *Mycobacterium tuberculosis* (P9WJ63) and *Brachyspira* (formerly *Serpulina*) *hyodysenteriae* (Q06803) TlyA sequences as queries (Monshupanee et al., 2012). Sequences were aligned with Clustal Omega (Sievers et al., 2011). Modeling of the tertiary structure of the Cj0588 catalytic domain was carried out with Modeller version 9.19 (Sali and Blundell, 1993) in UCSF Chimera (Pettersen et al., 2004) using *M. tuberculosis* TlyA (PDB: 5EOV) (Witek et al., 2017) as a reference to fold the Cj0588 peptide chain. The HhaI methyltransferase structure (PDB: 2HMY) was used to superimpose the AdoMet cofactor (O'Gara et al., 1999).

### Bacterial strains, plasmids, media, and growth conditions

The bacterial strains and plasmids used in this study are listed in Table S1. *C. jejuni* strains were grown at 37°C on Mueller-Hinton (MH, bioMérieux) agar containing 5% (v/v) sheep blood under microaerobic conditions using BD GasPak EZ CO_2_ sachets (Becton Dickinson). *Escherichia coli* strains were grown at 37°C in Luria Bertani (LB) broth or agar (bioMérieux) supplemented with chloramphenicol at 20 μg ml^−1^ and/or kanamycin at 25 μg ml^−1^.

### Transformation of *C. jejuni*

*Campylobacter jejuni* electrocompetent cells (Wassenaar et al., 1993) were mixed with plasmid DNA (0.5–5 μg) and electroporated using an Electro Cell Manipulator (ECM 600) at 50 μF, 126 Ω and 1.25 kV. Transformants were grown under microaerobic conditions at 37°C on MH plates for 5 h and then replated and grown for a further 2–5 days on MH agar containing 5% (v/v) sheep blood supplemented with chloramphenicol at 20 μg ml^−1^ and/or kanamycin at 25 μg ml^−1^.

### Construction and complementation of *C. jejuni cj0588*-null strains

Null mutants of the *C. jejuni* strains 405 and 81-176 were created by inserting a chloramphenicol *cat* resistance cassette (Cm^r^) into *cj0588* (*tlyA*). Briefly, the *cj0588* gene and flanking regions were amplified by PCR using the C5F and C5R primers (Table S2) and cloned into the pBluescript II SK plasmid; 450 bp of the *cj0588* coding sequence was then replaced with a *cat* cassette. The plasmids lack a *C. jejuni* replicon, and thus retention of Cm^r^ after electrotransformation of the *C. jejuni* strains requires homologous allelic exchange with the chromosomal *cj0588* gene. Recombinant plasmid structures were tested at each stage by restriction digestion; in all cases, plasmids and strains (Table S1) were finally verified by PCR sequencing (Table S2).

The *C. jejuni* 405Δ*cj0588* null-mutant was complemented *in trans* with plasmid-encoded versions of the *cj0588* gene. A fragment containing the *cj0588* gene was amplified by PCR from *C. jejuni* 81-176 using primers C2 and C5R (Table S2) and ligated into the *Xba*I-digested shuttle vector p0183 under *C. jejuni* promoter control to form plasmid pMW0588 (Salamasznska-Guz et al., 2013). This plasmid was used to transform *E. coli* before being moved to *C. jejuni* 405 forming strain 405Δ*cj0588*+pMW0588 (Table S1). The *C. jejuni* 81-176 null-mutant was complemented by inserting a wild-type copy *cj0588* under *C. jejuni* promoter control into the 121-bp intergenic region between *cj0652* and *cj0653c* (Javed et al., 2012) forming the *C. jejuni* strain 81-176Δ*cj0588*::*0588* (Table S1). Strains of 81-176 expressing the mutant versions of *cj0588* (described below) were created in the same way.

### Mutagenesis of the Cj0588 active site

The *cj0588* gene was mutagenized in plasmids pET0588 and pMW0588 using PCR primers (Table S2) and the QuikChange II (Agilent) protocol to individually substitute the K^80^, D^162^, and K^188^ codons with alanine codons.

Minimal inhibitory concentrations (MICs) were determined by diluting overnight cultures of *C. jejuni* strains and plating on MH sheep blood agar with 2-fold steps in drug concentration. MIC values are the lowest concentration at which no growth was observed after incubation under microaerobic conditions for 48 h at 37°C.

Polysome profiles of the *C. jejuni* Cj0588 recombinants were determined on sucrose gradients (Douthwaite et al., 1989) under nonstringent salt conditions [20 mM Tris-HCl pH 7.5, 60 mM NH_4_Cl, 10.5 mM Mg(CH_3_COO)_2_, 0.1 mM EDTA, 2 mM β-mercaptoethanol].

### Methylation of rRNA *in vivo*

Ribosomal subunits were prepared from *C. jejuni* strains on sucrose gradients for isolation of rRNAs, as described previously for *E. coli* (Douthwaite et al., 1989). 5′-^32^P-end-labeled deoxynucleotide primers were hybridized to complementary regions of 16S rRNA nucleotides 1411–1429 and 23S rRNA nucleotides 1926–1943, extended with AMV reverse transcriptase (Roche) and run on polyacrylamide/urea gels to detect sites of 2′-*O*-methylation (Maden et al., 1995; Johansen et al., 2006).

In MALDI-ToF (matrix assisted laser desorption-ionization time-of-flight) mass spectrometry analyses, the oligodeoxynucleotides SR199 and SJ20 (Table S2) were used to assess methylation in the *C. jejuni* rRNAs (Andersen et al., 2004; Douthwaite and Kirpekar, 2007). MALDI-ToF mass spectrometry data for rRNA samples fragmented with RNase A or T1 were collected in positive mode (UltrafleXtreme, Bruker).

### Analysis of *in vitro* methylation of rRNA

Histidine-tagged versions of the Cj0588 methyltransferase (Table S1) were purified from recombinant *E. coli* BL21(DE3) cells (Salamaszynska-Guz and Klimuszko, 2008). Methylation of rRNA by 250–500 nM purified Cj0588 protein was assayed in 50 mM Tris-Cl, pH 7.5, 10 mM EDTA, 10 mM β-mercaptoethanol, and 25 mM NaCl. The *K*_*m*_ for *S*-adenosyl-L-methionine (AdoMet) was determined using 6 μM 50S ribosomal subunits as substrate and increasing amounts of [^3^H]-AdoMet (10.0 Ci mmol^−1^ = 3.7 × 10^11^ Bq mmol^−1^; Amersham Biosciences). The *K*_*m*_ for the substrate was determined varying the concentration of 50S ribosomal subunits in the presence of 10 μM AdoMet for 30 min at 37 °C. The components were then spotted onto DE81 filter paper discs (Whatman, Brentford, UK) followed by air-drying and washing three times for 10 min with 50 mM KH_2_PO_4_, once with water, and finally with 70% ethanol. Incorporation of ^3^H-labeled methyl was measured by liquid scintillation (Wallace, Pharmacia), and for kinetic analysis Orgin software was used (OrginLab).

### Motility and biofilm assays

The motility of *C. jejuni* cells was followed by adding 3 μl of culture (OD_600_ 0.5) onto MH medium with 0.25% agar. Plates were left to dry and were incubated under microaerobic conditions for 48 h at 37°C before measuring cell migration.

Biofilm formation by the *C. jejuni* 405 and 81-176 strains was first assessed by staining. Strains were grown overnight in Mueller-Hinton broth at 37°C under microaerobic conditions in 24-well polystyrene plates. The broth was removed and cells were stained with 0.1% crystal violet for 5 min at room temperature. Unbound stain was removed by washing with water, and samples were dried and decolorized with ethanol/acetone before measuring absorbance at 570 nm using a BioMate spectrophotometer (Thermo Scientific).

Independent visualization of biofilms was carried out by Field Emission Scanning Electron Microscopy (FESEM). *C. jejuni* 81-176 recombinants were grown on Columbia agar plates for 24 h before harvesting and suspending in 5 ml BHI broth (OD_600_ = 0.05) and cultivating for 48 h at 37°C in 5% CO_2_ on glass cover slides. Biofilms were fixed for 24 h in 0.1 M cacodylate buffer (pH 7.3) with 3% glutaraldehyde followed by washing for 60 min in cacodylate buffer without glutaraldehyde, and then four times for 30 min in fresh buffer followed by dehydration for 6 h in 96% ethanol. Biofilms were air-dried and coated with gold-palladium (2–4 nm thick) and analyzed at nanometer image resolution by FESEM (MERLIN Carl Zeiss Germany) at 2–5 kV range accelerating voltage.

## Results

### The *C. jejuni* Cj0588 protein is a TlyA^I^ methyltransferase

**Figure 1:**
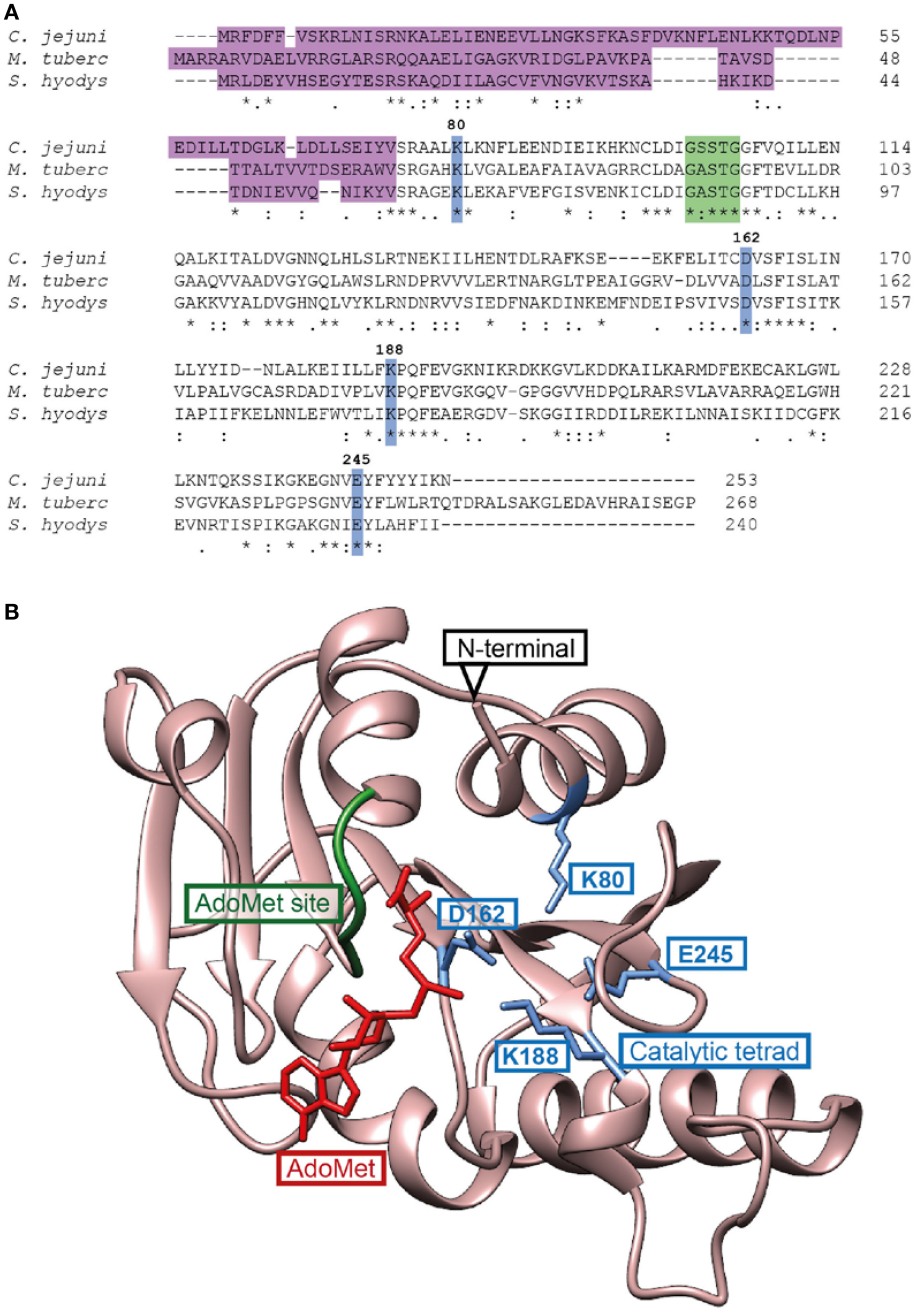
Structure of the *C. jejuni* TlyA methyltransferase homolog Cj0588. **(A)** Multiple alignment of the *C. jejuni* TlyA homolog Cj0588 (A0A0M4TPK5) with the *M. tuberculosis* type II TlyA (P9WJ63) and the *S. hyodysenteriae* (Q06803) type I TlyA sequences. The four extra residues at the N-terminus and the C-terminal extension are hallmarks of TlyA^II^ enzymes that modify 16S rRNA nucleotide C1409 in addition to 23S rRNA C1920 (Monshupanee et al., 2012). The S4 RNA binding domain of the proteins is shown in magenta. Key amino acid residues within the catalytic center are highlighted in blue (Cj0588 residue numbering), and a portion of the AdoMet cofactor binding site is shown in green. Highly conserved residues are indicated with asterisks. Sequences were aligned with Clustal Omega (Sievers et al., 2011). **(B)** Model of the catalytic domain of the Cj0588 structure based on the 1.7 Å resolution crystal structure of the *M. tuberculosis* TlyA (PDB: 5EOV) (Witek et al., 2017). The N-terminal S4 RNA binding domain is missing in the structure (Witek et al., 2017). The catalytic tetrad (blue); in this study, three of these residues, K80, D162 and K188, were individually substituted with alanine. The AdoMet cofactor (red) was positioned alongside residues in the highly conserved AdoMet site (green) using the HhaI methyltransferase structure (PDB: 2HMY) (O'Gara et al., 1999).

The sequence of the Cj0588 protein indicates that it belongs to the TlyA^I^ group, the members of which have a single methylation target on the 2′-*O*-position of 23S rRNA nucleotide C1920. In comparison with the orthologous group of TlyA^II^-type enzymes that methylate not only C1920 but also nucleotide C1409 in 16S rRNA (Monshupanee et al., 2012), Cj0588 lacks four amino acids (A2-R3-R4-A5) at its N–terminus and about twenty amino acids at its C–terminus (Figure 1A). Three-dimensional modeling of the catalytic domain of Cj0588 (Figure 1B) reveals a structure typical for 2′-*O*-methyltransferases with a seven-stranded β-sheet between five α-helix layers and four residues K^80^, D^162^, K^188^ and E^245^ that comprise the catalytic center (Arenas et al., 2011; Salamaszynska-Guz et al., 2014).

**Figure 2:**
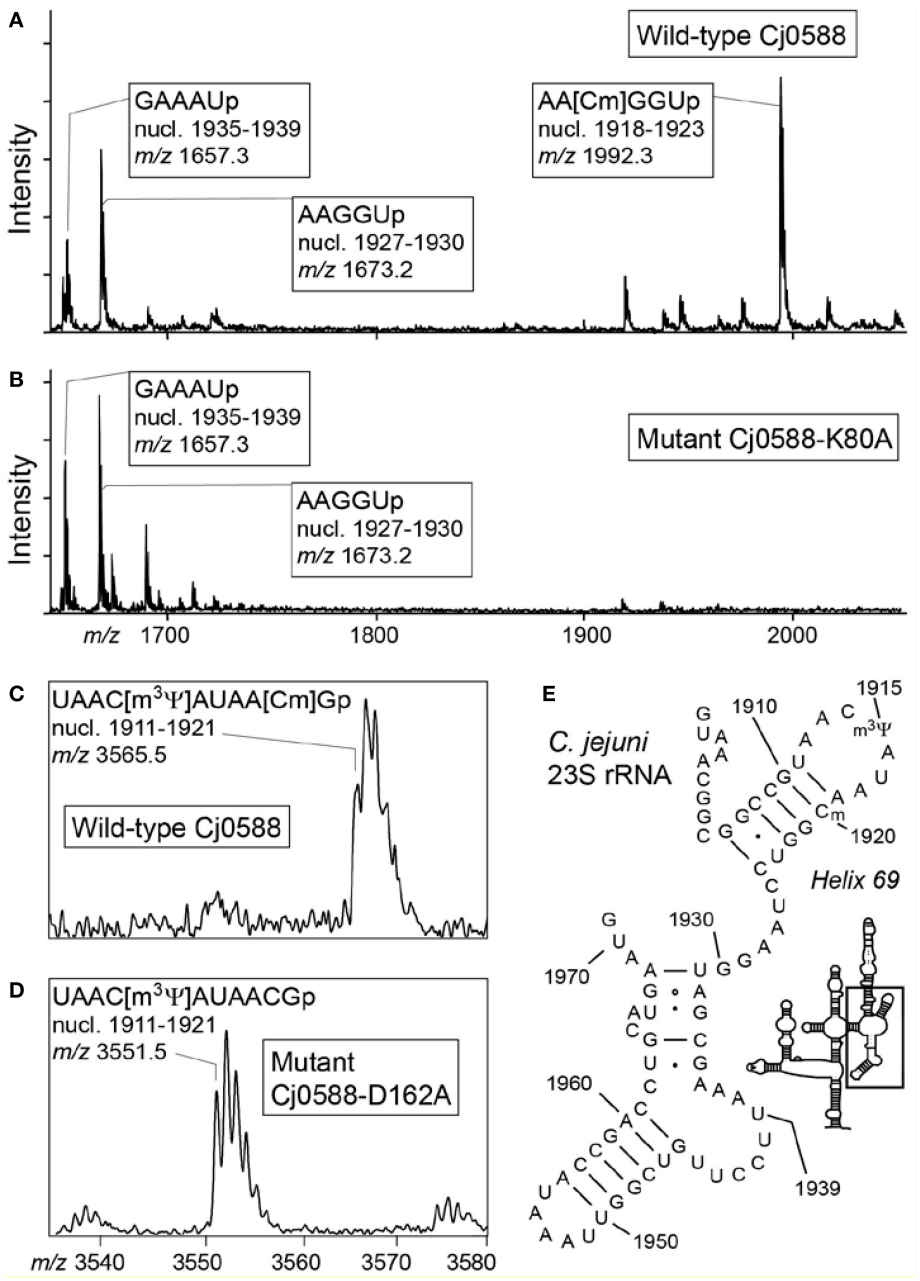
Mass spectrometric analyses of *C. jejuni* 23S rRNA. **(A)** Spectrum of RNase A fragments derived from the 50-nucleotide sequence encompassing C1920 in the 23S rRNA from the *C. jejuni* 405 wild-type strain. Nucleotide C1920 is evident in the AA[Cm]GGUp fragment at mass/charge (*m/z*) 1992, where 2′-*O*-methylation prevents RNase A cleavage. **(B)** This hexamer sequence is unmethylated in rRNA from the Cj0588-K80A mutant and is consequently cleaved to smaller AACp and GGUp fragments. The D162A and K188A strains produced fragmentation patterns identical to K80A (not shown). **(C)** After RNase T1 digestion, Cm1920 appears in the 11-mer fragment of nucleotides 1911–1921 together with nucleotide m^3^ψ1915. **(D)** The corresponding fragment from the Cj0588-D162A mutant showed that a single methyl group was missing. The K80A and K188A mutants (not shown) produced spectra identical to D162A. **(E)** Helix 69 and surrounding structures in the 23S rRNA (boxed in the schematic) including the sequence that was analyzed by MS. The m^5^U1939 modification, which is present in most bacteria (Desmolaize et al., 2011), is missing in *C. jejuni* (the unmodified nucleotide is evident in the GAAAUp fragment in **A,B**).

**Figure 3:**
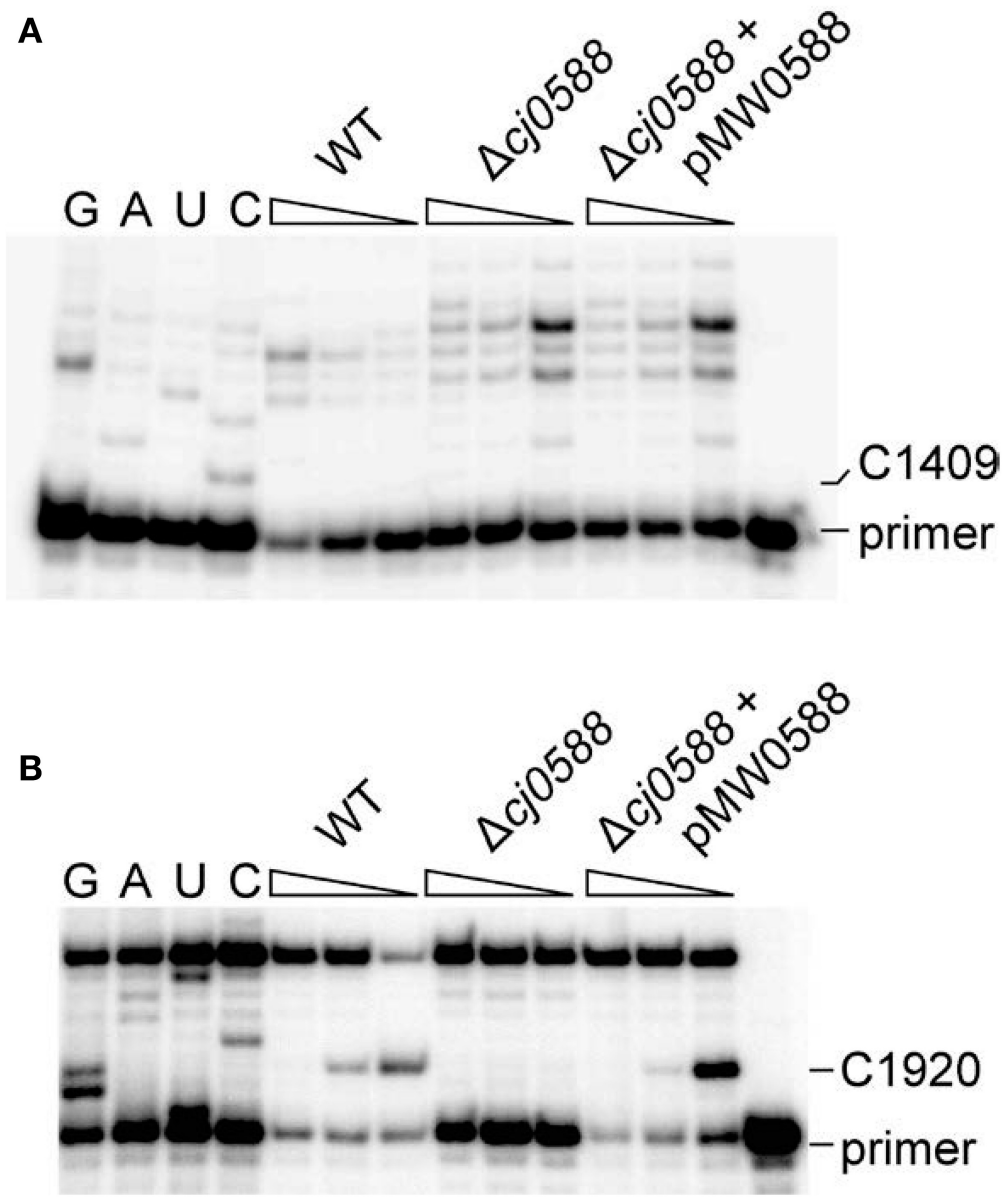
*In vivo* activity of the *C. jejuni* Cj0588 methyltransferase. Gel autoradiograms of primer extensions on **(A)** 16S rRNA and **(B)** 23S rRNA from *C. jejuni* 405 strains: WT, wild-type; Δ*cj0588*, null-strain; Δc*j0588*+pMW0588, null-strain complemented with an active, plasmid-encoded copy of c*j0588*. Decreasing dGTP concentrations (indicated by wedges) intensifies reverse transcription pausing at cytidines with 2′-*O*-methylation (Maden et al., 1995). Methylation by Cj0588 is specific for 23S rRNA nucleotide C1920. Lanes G, A, U, and C are dideoxy sequencing reactions on unmodified *C. jejuni* rRNAs.

The predictions about the specificity of nucleotide methylation were tested by analyzing the rRNAs of *C. jejuni* 405 and 81-176 strains with and without the Cj0588 protein. MALDI-ToF mass spectrometric analysis of 16S rRNA from wild-type strains showed that there was no methylation at nucleotide C1409 (Figure S1), whereas a clear top was seen corresponding to the fragment containing 2′-*O*-methylation at 23S rRNA nucleotide C1920 (Figure 2A). These findings were confirmed by primer extension (Figure 3). As anticipated, there was no methylation at these nucleotides in the *cj0588-*null mutants, and methylation capacity was restored by complementation of null-mutants with an intact copy of the *C. jejuni cj0588* gene (Figure 3). The rRNA methylation analyses together with the *in silico* studies of the *C. jejuni* Cj0588 structure establish that this protein belongs to the TlyA^I^ group of RNA methyltransferases that modify solely at 23S rRNA nucleotide C1920.

### Additional modifications within antibiotic binding regions of *C. jejuni* rRNAs

The mass spectrometry data revealed several additional modifications within helix 44 of *C. jejuni* 16S rRNA and helix 69 of 23S rRNA, where interactions with diverse groups of antibiotics occur (Johansen et al., 2006; Wilson, 2009). In 16S rRNA, two methyl groups on nucleotide C1402 and an additional methylation at C1404 were evident (Figures S1A,B), and there were no further modifications at other nucleotides in this sequence. These 16S rRNA methylations are presumably added by enzymes encoded by the *C. jejuni* genes *cj0636, cj0693c*, and *cj0154c* that are homologous to the *E. coli* rRNA methyltransferase genes *rsmF, rsmH*, and *rsmI* (Figure S1E). The *E. coli* RsmH and RsmI enzymes respectively methylate the base and ribose of C1402 (Kimura and Suzuki, 2010), and have 32 and 35% identity with the homologous *C. jejuni* proteins. The m^5^C1404 modification is presumably added by Cj0636, which is orthologous to NOL1/NOP2/sun proteins including the *Enterococcus faecium* methyltransferase (35% identical) that modifies the same 16S rRNA nucleobase (Galimand et al., 2011), and *E. coli* RsmF that methylates the 5-position of the neighboring nucleotide C1407 (Andersen and Douthwaite, 2006).

In 23S rRNA, U1915 is converted to m^3^ψ (Figure 2C) by the enzymes encoded by the *C. jejuni* genes *cj1280c* and *cj0126c* that are equivalent to the *E. coli* pseudouridine synthetase RluD (Huang et al., 1998; Wrzesinski et al., 2000) and methyltransferase RlmH (Ero et al., 2008; Purta et al., 2008), respectively. No modification was observed at nucleotide U1939 (Figure 2A), which is commonly methylated in many bacteria (Agarwalla et al., 2002; Madsen et al., 2003), and consistent with this, no homolog of the *rlmD* gene that encodes the m^5^U1939 methyltransferase enzyme is evident in the *C. jejuni* genome.

### Substitutions in the Cj0588/TlyA catalytic center eliminate methylation activity

**Figure 4:**
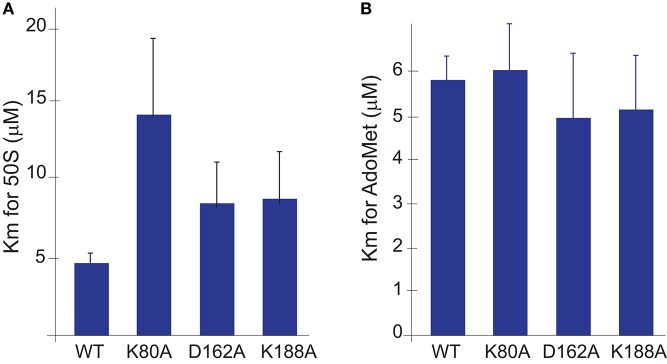
Binding affinities of the Cj0588 protein variants measured *in vitro* with **(A)** the 50S ribosomal subunit substrate and **(B)** the methyl-donor cofactor, AdoMet. Values represent means and standard errors of three independent experiments.

Three of the four key residues in the catalytic tetrad were individually substituted with alanine to elimated methyltransferase activity without perturbing the overall structure of the protein. Assaying the *C. jejuni* mutant Cj0588 enzymes under *in vitro* conditions where the substrate and cofactor were present in saturating amounts showed that binding affinity for the AdoMet cofactor was not significantly altered by the K80A, D162A, and K188A substitutions (Figure 4). These substitutions had a mild effect on the binding affinity for the 50S subunit substrate, increasing the K_m_ between 2- and 3-fold (Figure 4).

Expression of the mutant Cj0588 enzymes *in vivo* in *C. jejuni* followed by rRNA analysis by mass spectrometry and primer extension showed that each of the K80A, D162A and K188A substitutions led to complete loss of rRNA methylation (Figure 2, Figure S2). The Cj0588 proteins behaved in an identical manner when expressed in *E. coli* where the wild-type enzyme methylated effectively at 23S rRNA C1920, and with no methylation at this nucleotide in strains expressing the mutant enzymes (data not shown).

**Figure 5:**
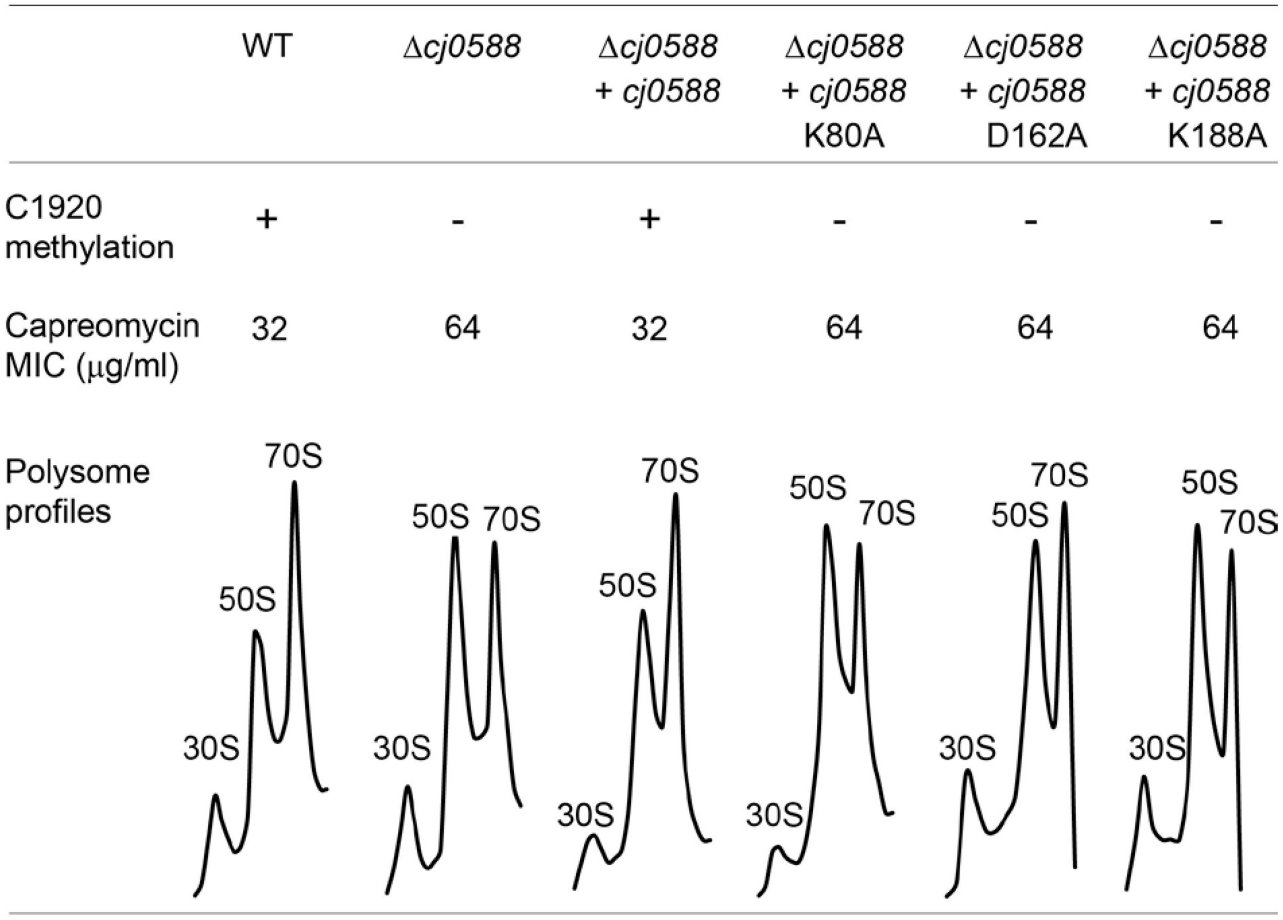
Effect of Cj0588 methyltransferase activity on capreomycin tolerance and ribosomal subunit association in *C. jejuni* strain 405. Positions and relative proportions of 30S and 50S ribosomal subunits and 70S ribosomes are indicated on sucrose gradient profiles.

In *M. tuberculosis*, loss of ribose methylation at nucleotide C1920 and/or C1409 affects the interaction between capreomycin and the ribosome sufficiently to confer clinical resistance (Maus et al., 2005; Johansen et al., 2006). In *C. jejuni*, loss of Cj0588-directed methylation increases the MIC of capreomycin by 2-fold and interferes with the association of ribosomal subunits to form functional 70S ribosomes (Figure 5), although this was not accompanied by significant slowing of cell growth under the conditions assayed here (Figure S3). The distribution of ribosome particles was rescued by complementation of the null strains with a functional *cj0588* gene. However, complementation with the K80A, D162A, and K188A mutant variants of *cj0588* did not restore the ribosome profiles (Figure 5), confirming that Cj0588 methylation facilitates the association of the ribosomal subunits.

### Inactivation of Cj0588 hinders *C. jejuni* motility

**Figure 6:**
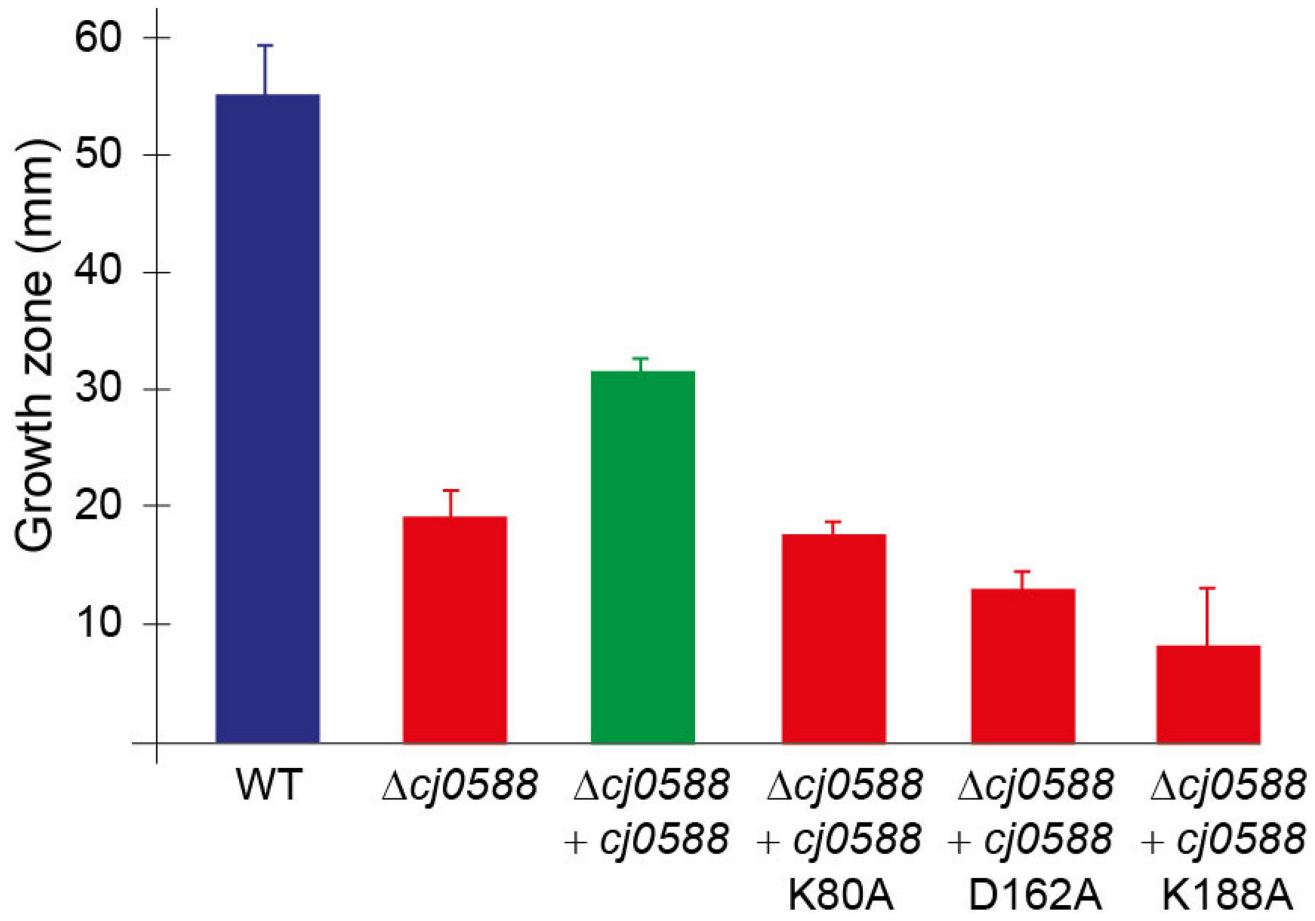
The motility of the *C. jejuni* 405 wild-type strain (WT) was reduced from a growth zone of 54.5 ± 5.4 mm to 19.1 ± 2.7 mm after loss of *cf0588* function (Δ*cj0588*). Complementation of the null mutant with a plasmid-encoded, active copy of the *cj0588* gene (Δ*cj0588*+*cj0588*) partially restored motility to 59% (32 ± 1.2 mm). However, complementation of the null-strain with the K80A, D162A and K188A mutant variants did not rescue this function. The *C. jejuni* 81-176 strain showed the same dependence on Cj0588 activity for motility. The 81-176 strain was generally less motile under these conditions, with a wild-type growth zone of 24.3 ± 8.6 mm, reduced to 8.7 ± 4.2 mm for the *cj0588*-null strain. Complementation by inserting an active *cj0588* gene into the chromosome of the 81-176 null strain restored motility to 66% (16.1 ± 3.5 mm), whereas inactive gene copies did not rescue the phenotype. Values represent means ± S.E.M. of three independent experiments. *P* < 0.001 for WT vs. Δ*cj0588*; and *P* < 0.01 for Δ*cj0588* vs. Δ*cj0588*+*cj0588*.

Inactivation of *cj0588* in both the *C. jejuni* 405 and 81-176 strains reduced motility by about 65%, which is consistent with our earlier observations for the 81-176 strain (Salamaszynska-Guz et al., 2014). We continued the motility studies using the 405 strain after establishing that its growth zones on agar were greater than those of the 81-176 strain (Figure 6). Complementing the 405-null strain with an active copy of *cj0588* partially restored motility, whereas complementation with the methyltransferase deficient K80A, D162A and K188A variants did not significantly rescue the *cj0588-*null phenotype (Figure 6).

### Biofilm formation by *C. jejuni* is impaired after inactivation of *cj0588*

*Campylobacter jejuni* forms biofilms on abiotic surfaces such as polystyrene or glass (Joshua et al., 2006; Kalmokoff et al., 2006; Reeser et al., 2007) as well as on living tissue (Epps et al., 2013; Luethy et al., 2017). The *C. jejuni* 81-176 strain is particularly proficient at attaching to abiotic surfaces (Turonova et al., 2015) in addition to forming aggregates in liquid and biofilms in the human intestinal tract (Joshua et al., 2006; Gunther and Chen, 2009; Haddock et al., 2010). In crystal violet staining assays *in vitro*, the *C. jejuni* 81-176 strain was found to be significantly more effective than strain 405 at forming biofilms on both polystyrene (A_570_ = 0.53 ± 0.17 vs. 0.26 ± 0.05) and on glass. Our subsequent biofilm studies thus focused on the 81-176 strain.

**Figure 7:**
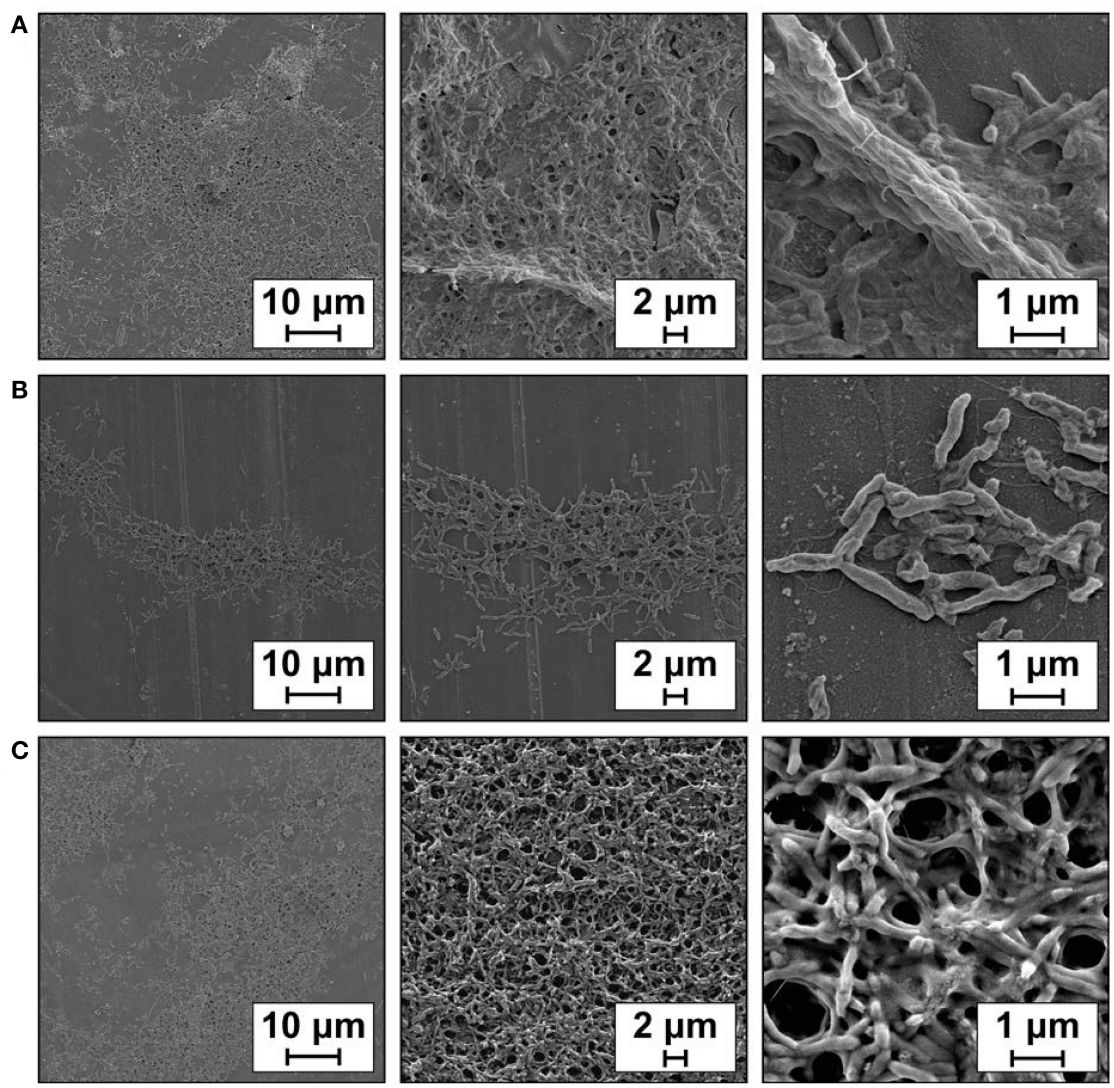
Biofilm produced by *C. jejuni* 81-176 on cover glass after 48 h at 37°C under microaerobic conditions and visualized by Field Emission Scanning Electron Microscopy. **(A)** *C. jejuni* 81-176 wild-type, **(B)** *C. jejuni* 81-176Δ*cj0588*, **(C)** the complemented *C. jejuni* strain 81-176Δ*cj0588*::*0588*. Experiments were performed in triplicate and representative micrographs are shown.

**Figure 8:**
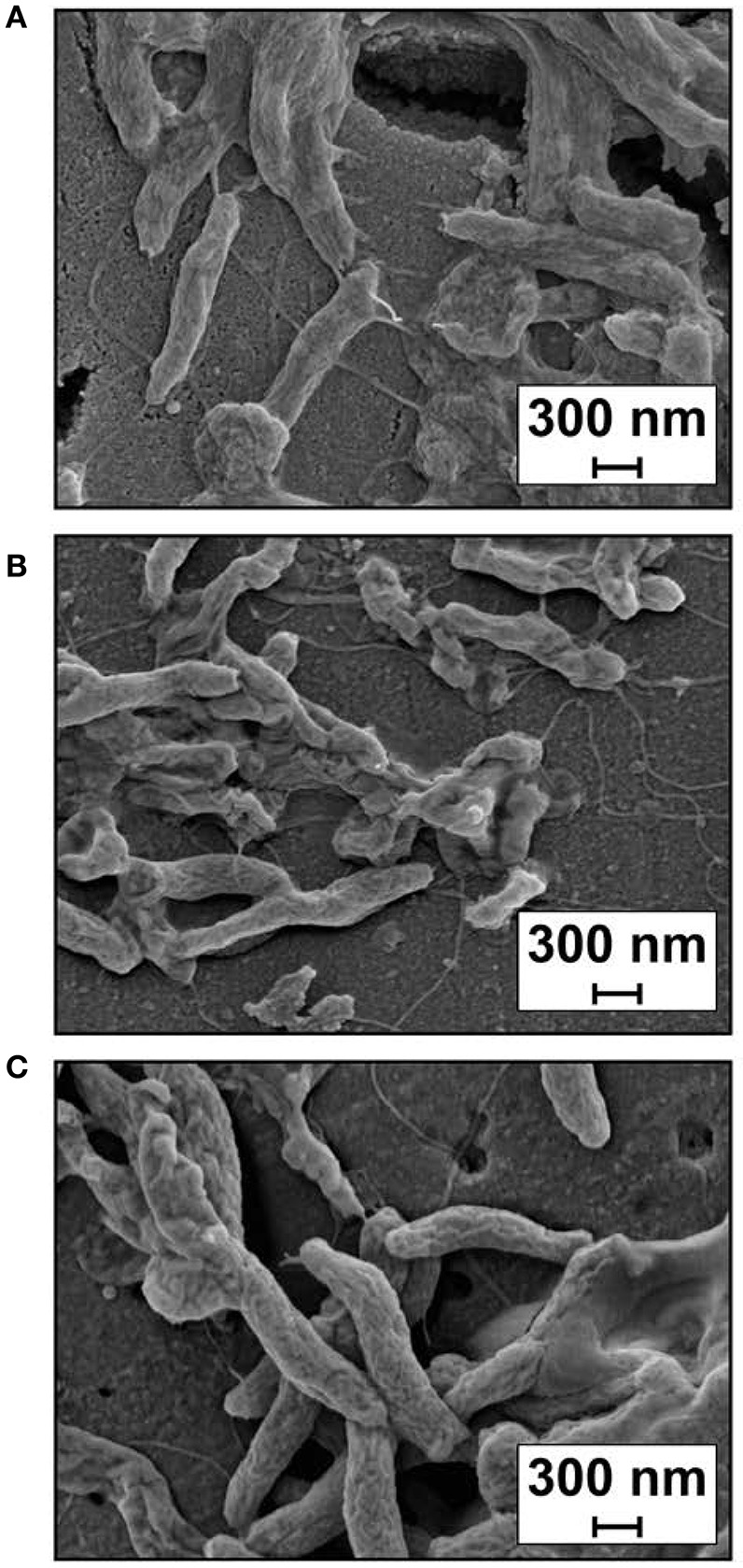
Cell morphology from areas of the electromicrographs where individual cells can be distinguished. **(A)** *C. jejuni* 81-176 wild-type strain, **(B)** *C. jejuni* 81-176Δ*cj0588*, and **(C)** the complemented strain *C. jejuni* 81-176Δ*cj0588*::*0588*. Experiments were performed in triplicate and representative micrographs are shown.

A clear visualization of biofilm formation was attained using Field Emission Scanning Electron Microscopy (FESEM) and showed that wild-type *C. jejuni* 81-176 cells adhered together to cover the glass almost entirely after 48 h growth (Figure 7A). In contrast, the biofilm formed by the *cj0588-*null strain (Figure 7B) was less extensive, with cells forming clumps lacking the structural roughness of the wild-type strain. These morphological differences were more evident in the enlarged views of individual cells (Figure 8) where the null-mutant cells are clearly less bulky, which would indicate that they have secreted reduced quantities of the extracellular matrix required for biofilm formation. Apart from this difference, the mutant cell images revealed no other obvious variation in dimensions, shape or flagellar structure that might relate to their reduced motility and capacity to form biofilms (Figure 8).

The biofilm forming capacity of the null-mutant was partially restored after being complemented with a functional copy of the *cj0588* gene (Figure 7C), and this correlated with cells regained their bulky appearance (Figure 8C). The inactive versions of the *cj0588* gene did not rescue these morphological features, and cells continued to produce biofilms that were less copious than the null-mutant complemented with the active *cj0588* gene (Figure S4).

## Discussion

We show here that the *cj0588* gene of *C. jejuni* encodes a type I TlyA methyltransferase that specifically targets the 2′-*O*-ribose position of 23S rRNA nucleotide C1920, but does not modify 16S rRNA nucleotide C1409. This puts the *C. jejuni* Cj0588 enzyme in the same class as other TlyA^I^ methyltransferases from *B. hyodysenteriae* and *T. thermophilus*, and distinguishes it from the TlyA^II^ enzymes of *Mycobacterium* and *Streptomyces* species that methylate the ribose of 16S rRNA nucleotide C1409 and also at C1920 (Monshupanee et al., 2012). Both types of TlyA enzyme belong to the cluster of orthologous groups COG2933 and are *S*-adenosylmethionine-dependent, Rossmann-fold methyltransferases related to small RNA-guided fibrillarins found in the Archaea and Eukaryota (Feder et al., 2003). These methyltransferases are characterized by a catalytic tetrad of sequence motifs centered around the conserved amino acid residues K-D-K-E, where three of these residues correspond to K^80^, D^162^ or K^188^ in the *C. jejuni* Cj0588 enzyme (Figure 1).

Substitution of the equivalent lysine residues in the *E. coli* methyltransferase RrmJ significantly decreased its ability to 2′-*O*-methylate 23S rRNA nucleotide U2552 without affecting the K_m_ for the enzyme-substrate interaction (Hager et al., 2002). Similarly, each of the K80A, D162A and K188A substitution in Cj0588 resulted in complete loss of methylation at the target nucleotide C1920 in *C. jejuni* (Figure 2, Figure S3). Assays with recombinant Cj0588 proteins that were expressed and purified from *E. coli* showed that cofactor binding was not affected by the K80A, D162A and K188A substitutions and that there was only a moderate reduction in substrate binding (Figure 4). These observations indicate that the substitutions removed the methyltransferase activity of Cj0588 without interfering with its synthesis or its overall tertiary structure. These constructions enabled us to differentiate between effects caused by Cj0588/TlyA-directed rRNA methylation and other putative functions in bacterial virulence that this enzyme might have.

The Cj0588 protein was indeed shown to be linked with an array of additional functions. Loss of Cj0588 reduces the ability of the ribosomal subunit to form a stable 70S complex (Figure 5), increases tolerance to the cyclic peptide antibiotic capreomycin (Figure 5), reduces motility (Figure 6), and diminishes biofilm formation (Figure 7) while decreasing cell bulkiness (Figure 8). These properties are to a large extent restored to wild-type levels by complementing the *C. jejuni* null-strains with an active copy of *cj0588*, and can thus be concluded to be dependent on the presence of the Cj0588 protein.

The more specific question of whether the rRNA methylation capability of Cj0588 promotes these effects was addressed by further rounds of null-strain complementation with the methyltransferase-inactive K80A, D162A, and K188A constructs. For the most part, the conclusions are clear: the phenotypic changes are caused primarily by loss of rRNA methylation and could not be rescued by the catalytically inactive variants of Cj0588. Formation of stable 70S ribosomes and maintenance of capreomycin susceptibility require rRNA methylation. The 23S rRNA nucleotide C1920 is located at the interface where the ribosomal subunits make contact at interbridge B2a (Yusupov et al., 2001; Liu and Fredrick, 2016), which also encompasses the capreomycin binding site (Johansen et al., 2006; Stanley et al., 2010), and C1920 methylation directly affects these processes. Efficient motility of the *C. jejuni* strains also required Cj0588 methyltransferase activity and motility was not restored to wild-type levels by the catalytically defective versions of this protein. These effects thus result from a change in the rRNA methylation at the ribosomal subunit interface, with reduced motility being one of the consequences of altered ribosome function and protein synthesis.

The ability to form biofilms is a defining feature of *C. jejuni* virulence with surface attachment being an essential step in intestinal colonization and infection (Haddock et al., 2010; Epps et al., 2013). It has previously been shown that mutation in the *cj0588*/*tlyA* gene of *C. jejuni* reduces its colonization of the Caco-2 epithelial cell line (Salamaszynska-Guz and Klimuszko, 2008). Furthermore, mutation of the *tlyA* homolog in *B. hyodysenteriae* reduces virulence, while loss of *tlyA* function in *Helicobacter pylori* lowers adhesion to human gastric adenocarcinoma cells and prevents colonization of the gastric mucosa of mice (Hyatt et al., 1994; Martino et al., 2001; Zhang et al., 2002). Consistent with these previous observations, we show here that loss of its Cj0588 enzyme results in a significant reduction in the ability of *C. jejuni* to adhere to surfaces *in vitro* (Figure 7). A degree of rescue of the *C. jejuni* biofilm phenotype was obtained by complementation with an active *cj0588* gene, and improvement of the phenotype was clearly better than complementation with inactivated copies of the gene (Figure S4).

Enigmatically, biofilm formation by *C. jejuni* requires flagella expression (Guerry, 2007; Svensson et al., 2014) although motile strains do not necessarily form biofilms (Brown et al., 2015). All the *C. jejuni cj0588*-null strains studied here possess flagella (Figure 8) and could to some degree adhere to surfaces (Figure 7B, Figure S4). The *C. jejuni* 405 adhered less well than the slower 81-176 strain to plastic and glass surfaces, consistent with traits other than motility being crucial for biofilm formation. The methyltransferase-deficient cells were less bulky (Figure 8) and tended to form clumps without developing into the biofilm structures observed for wild type cells and the *cj0588*-complemented cells (Figure 7). In these latter cells, the biofilm phenotype was not fully rescued and the reason for this is not clear. Possibly, removal of *cj0588* and its reintroduction at another chromosomal location could effect changes in the expression of other genes connected with biofilm formation.

Loss of other rRNA modifications have previously been noted to cause a series of pleiotropic effects and result in loss of virulence in pathogenic bacteria (Sergiev et al., 2011). We show here that loss of the TlyA enzyme homolog Cj0588 in *C. jejuni*, results in defective ribosome subunit association, reduced motility and biofilm formation, and altered sensitivity to the antibiotic capreomycin. Complementation with methyltransferase-defective variants of Cj0588 show that all these effects are related to loss of rRNA methylation rather than loss of the protein itself. From this, it can be concluded that the manner in which Cj0588 is connected with the physiology or pathogenicity of *C. jejuni* is solely a consequence of its rRNA methylation activity. Changes in subunit association and capreomycin sensitivity would result directly from altered ribosomal interactions at the site of the (missing) methylation (Johansen et al., 2006). The phenotypic defects including reduced motility and biofilm formation would result from changes in protein expression on ribosomes lacking the nucleotide C1920 methylation. The exact nature of the proteome changes and how these are linked to loss of motility, biofilm formation and virulence remain to be determined.

## Author contributions

AS-G, SR, CL, PB, BT, and TU: Methodology and Investigation; SD and AS-G: Writing, Review and Editing; SD, PB, and AS-G: Resources; SD and AS-G: Funding Acquisition and Supervision.

### Conflict of interest statement

The authors declare that the research was conducted in the absence of any commercial or financial relationships that could be construed as a potential conflict of interest.

## References

[B1] Agarwalla S. Kealey J. T. Santi D. V. Stroud R. M. (2002). Characterization of the 23S ribosomal RNA m^5^U1939 methyltransferase from *Escherichia coli*. J. Biol. Chem. 277, 8835–8840. 10.1074/jbc.M111825200 11779873

[B2] Altschul S. F. Madden T. L. Schäffer A. A. Zhang J. Zhang Z. Miller W.. (1997). Gapped BLAST and PSI-BLAST: a new generation of protein database search programs. Nucleic Acids Res. 25, 3389–3402. 10.1093/nar/25.17.3389 9254694 PMC146917

[B3] Andersen N. M. Douthwaite S. (2006). YebU is a m^5^C methyltransferase specific for 16S rRNA nucleotide 1407. J. Mol. Biol. 359, 777–786. 10.1016/j.jmb.2006.04.007 16678201

[B4] Andersen T. E. Porse B. T. Kirpekar F. (2004). A novel partial modification at 2501 in *Escherichia coli* 23S ribosomal RNA. RNA 10, 907–913. 10.1261/rna.5259404 15146074 PMC1370582

[B5] Arenas N. E. Salazar L. M. Soto C. Y. Vizcaíno C. Patarroyo M. E. Patarroyo M. A.. (2011). Molecular modeling and *in silico* characterization of *Mycobacterium tuberculosis* TlyA: possible misannotation of this tubercle bacilli-hemolysin. BMC Struct. Biol. 11:16. 10.1186/1472-6807-11-16 21443791 PMC3072309

[B6] Bolton D. J. (2015). *Campylobacter* virulence and survival factors. Food Microbiol. 48, 99–108. 10.1016/j.fm.2014.11.017 25790997

[B7] Brown H. L. Reuter M. Hanman K. Betts R. P. Van Vliet A. H. (2015). Prevention of biofilm formation and removal of existing biofilms by extracellular DNases of *Campylobacter jejuni*. PLoS ONE 10:e0121680. 10.1371/journal.pone.0121680 25803828 PMC4372405

[B8] Bügl H. Fauman E. B. Staker B. L. Zheng F. Kushner S. R. Saper M. A.. (2000). RNA methylation under heat shock control. Mol. Cell 6, 349–360. 10.1016/S1097-2765(00)00035-6 10983982

[B9] Desmolaize B. Fabret C. Bregeon D. Rose S. Grosjean H. Douthwaite S. (2011). A single methyltransferase YefA (RlmCD) catalyses both m^5^U747 and m^5^U1939 modifications in *Bacillus subtilis* 23S rRNA. Nucleic Acids Res. 39, 9368–9375. 10.1093/nar/gkr626 21824914 PMC3241648

[B10] Douthwaite S. Kirpekar F. (2007). Identifying modifications in RNA by MALDI mass spectrometry. Method Enzymol. 425, 3–20. 10.1016/S0076-6879(07)25001-3 17673077

[B11] Douthwaite S. Powers T. Lee J. Y. Noller H. F. (1989). Defining the structural requirements for a helix in 23S ribosomal RNA that confers erythromycin resistance. J. Mol. Biol. 209, 655–665. 10.1016/0022-2836(89)93000-3 2685326

[B12] Epps S. V. Harvey R. B. Hume M. E. Phillips T. D. Anderson R. C. Nisbet D. J. (2013). Foodborne *Campylobacter*: infections, metabolism, pathogenesis and reservoirs. Int. J. Environ. Res. Public Health 10, 6292–6304. 10.3390/ijerph10126292 24287853 PMC3881114

[B13] Ero R. Peil L. Liiv A. Remme J. (2008). Identification of pseudouridine methyltransferase in *Escherichia coli*. RNA 14, 2223–2233. 10.1261/rna.1186608 18755836 PMC2553739

[B14] Feder M. Pas J. Wyrwicz L. S. Bujnicki J. M. (2003). Molecular phylogenetics of the RrmJ/fibrillarin superfamily of ribose 2′-*O*-methyltransferases. Gene 302, 129–138. 10.1016/S0378-1119(02)01097-1 12527203

[B15] Galimand M. Schmitt E. Panvert M. Desmolaize B. Douthwaite S. Mechulam Y.. (2011). Intrinsic resistance to aminoglycosides in *Enterococcus faecium* is conferred by the 16S rRNA m^5^C1404-specific methyltransferase EfmM. RNA 17, 251–262. 10.1261/rna.2233511 21159796 PMC3022275

[B16] Guerry P. (2007). Campylobacter flagella: not just for motility. Trends Microbiol. 15, 456–461. 10.1016/j.tim.2007.09.006 17920274

[B17] Gunther N. W. T. Chen C. Y. (2009). The biofilm forming potential of bacterial species in the genus Campylobacter. Food Microbiol. 26, 44–51. 10.1016/j.fm.2008.07.012 19028304

[B18] Haddock G. Mullin M. Maccallum A. Sherry A. Tetley L. Watson E.. (2010). *Campylobacter jejuni* 81-176 forms distinct microcolonies on *in vitro*-infected human small intestinal tissue prior to biofilm formation. Microbiology 156, 3079–3084. 10.1099/mic.0.039867-0 20616103

[B19] Hager J. Staker B. L. Bugl H. Jakob U. (2002). Active site in RrmJ, a heat shock-induced methyltransferase. J. Biol. Chem. 277, 41978–41986. 10.1074/jbc.M205423200 12181314

[B20] Huang L. Ku J. Pookanjanatavip M. Gu X. Wang D. Greene P. J.. (1998). Identification of two *Escherichia coli* pseudouridine synthases that show multisite specificity for 23S RNA. Biochemistry 37, 15951–15957. 10.1021/bi981002n 9843401

[B21] Hyatt D. R. Ter Huurne A. A. Van Der Zeijst B. A. Joens L. A. (1994). Reduced virulence of *Serpulina hyodysenteriae* hemolysin-negative mutants in pigs and their potential to protect pigs against challenge with a virulent strain. Infect. Immun. 62, 2244–2248. 8188345 10.1128/iai.62.6.2244-2248.1994 PMC186504

[B22] Javed M. A. Cawthraw S. A. Baig A. Li J. Mcnally A. Oldfield N. J.. (2012). Cj1136 is required for lipooligosaccharide biosynthesis, hyperinvasion, and chick colonization by *Campylobacter jejuni*. Infect. Immun. 80, 2361–2370. 10.1128/IAI.00151-12 22508861 PMC3416457

[B23] Johansen S. K. Maus C. E. Plikaytis B. B. Douthwaite S. (2006). Capreomycin binds across the ribosomal subunit interface using *tlyA*-encoded 2′-O-methylations in 16S and 23S rRNAs. Mol. Cell 23, 173–182. 10.1016/j.molcel.2006.05.044 16857584

[B24] Joshua G. W. Guthrie-Irons C. Karlyshev A. V. Wren B. W. (2006). Biofilm formation in *Campylobacter jejuni*. Microbiology 152, 387–396. 10.1099/mic.0.28358-0 16436427

[B25] Kalmokoff M. Lanthier P. Tremblay T. L. Foss M. Lau P. C. Sanders G.. (2006). Proteomic analysis of *Campylobacter jejuni* 11168 biofilms reveals a role for the motility complex in biofilm formation. J. Bacteriol. 188, 4312–4320. 10.1128/JB.01975-05 16740937 PMC1482957

[B26] Kimura S. Suzuki T. (2010). Fine-tuning of the ribosomal decoding center by conserved methyl-modifications in the *Escherichia coli* 16S rRNA. Nucleic Acids Res. 38, 1341–1352. 10.1093/nar/gkp1073 19965768 PMC2831307

[B27] Liu Q. Fredrick K. (2016). Intersubunit Bridges of the Bacterial Ribosome. J. Mol. Biol. 428, 2146–2164. 10.1016/j.jmb.2016.02.009 26880335 PMC4884490

[B28] Luethy P. M. Huynh S. Ribardo D. A. Winter S. E. Parker C. T. Hendrixson D. R. (2017). Microbiota-derived short-chain fatty acids modulate expression of *Campylobacter jejuni* determinants required for commensalism and virulence. MBio 8:e00407-17. 10.1128/mBio.00407-17 28487428 PMC5424204

[B29] Maden B. E. H. Corbett M. E. Heeney P. A. Pugh K. Ajuh P. M. (1995). Classical and novel approaches to the detection and localization of the numerous modified nucleotides in eukaryotic ribosomal RNA. Biochimie 77, 22–29. 10.1016/0300-9084(96)88100-4 7599273

[B30] Madsen C. T. Mengel-Jørgensen J. Kirpekar F. Douthwaite S. (2003). Identifying the methyltransferases for m^5^U747 and m^5^U1939 in 23S rRNA using MALDI mass spectrometry. Nucleic Acids Res. 31, 4738–4746. 10.1093/nar/gkg657 12907714 PMC169892

[B31] Martino M. C. Stabler R. A. Zhang Z. W. Farthing M. J. Wren B. W. Dorrell N. (2001). *Helicobacter pylori* pore-forming cytolysin orthologue TlyA possesses *in vitro* hemolytic activity and has a role in colonization of the gastric mucosa. Infect. Immun. 69, 1697–1703. 10.1128/IAI.69.3.1697-1703.2001 11179345 PMC98074

[B32] Maus C. E. Plikaytis B. B. Shinnick T. M. (2005). Mutation of *tlyA* confers capreomycin resistance in *Mycobacterium tuberculosis*. Antimicrob. Agents Chemother. 49, 571–577. 10.1128/AAC.49.2.571-577.2005 15673735 PMC547314

[B33] Monshupanee T. (2013). Increased bacterial hemolytic activity is conferred by expression of TlyA methyltransferase but not by its 2′-*O*-methylation of the ribosome. Curr. Microbiol. 67, 61–68. 10.1007/s00284-013-0332-7 23417025

[B34] Monshupanee T. Johansen S. K. Dahlberg A. E. Douthwaite S. (2012). Capreomycin susceptibility is increased by TlyA-directed 2′-*O*-methylation on both ribosomal subunits. Mol. Microbiol. 85, 1194–1203. 10.1111/j.1365-2958.2012.08168.x 22779429 PMC3438285

[B35] O'Gara M. Zhang X. Roberts R. J. Cheng X. (1999). Structure of a binary complex of HhaI methyltransferase with S-adenosyl-L-methionine formed in the presence of a short non-specific DNA oligonucleotide. J. Mol. Biol. 287, 201–209. 10.1006/jmbi.1999.2608 10080885

[B36] Pettersen E. F. Goddard T. D. Huang C. C. Couch G. S. Greenblatt D. M. Meng E. C.. (2004). UCSF Chimera–a visualization system for exploratory research and analysis. J. Comput. Chem. 25, 1605–1612. 10.1002/jcc.20084 15264254

[B37] Punekar A. S. Shepherd T. R. Liljeruhm J. Forster A. C. Selmer M. (2012). Crystal structure of RlmM, the 2′-*O*-ribose methyltransferase for C2498 of *Escherichia coli* 23S rRNA. Nucleic Acids Res. 40, 10507–10520. 10.1093/nar/gks727 22923526 PMC3488215

[B38] Purta E. Kaminska K. H. Kasprzak J. M. Bujnicki J. M. Douthwaite S. (2008). YbeA is the m^3^Ψ methyltransferase RlmH that targets nucleotide 1915 in 23S rRNA. RNA 14, 2234–2244. 10.1261/rna.1198108 18755835 PMC2553730

[B39] Reeser R. J. Medler R. T. Billington S. J. Jost B. H. Joens L. A. (2007). Characterization of *Campylobacter jejuni* biofilms under defined growth conditions. Appl. Environ. Microbiol. 73, 1908–1913. 10.1128/AEM.00740-06 17259368 PMC1828834

[B40] Reuter M. Mallett A. Pearson B. M. Van Vliet A. H. (2010). Biofilm formation by *Campylobacter jejuni* is increased under aerobic conditions. Appl. Environ. Microbiol. 76, 2122–2128. 10.1128/AEM.01878-09 20139307 PMC2849235

[B41] Salamasznska-Guz A. Grodzik M. Klimuszko D. (2013). Mutational analysis of *cj0183 Campylobacter jejuni* promoter. Curr. Microbiol. 67, 696–702. 10.1007/s00284-013-0420-8 23884593 PMC3824568

[B42] Salamaszynska-Guz A. Klimuszko D. (2008). Functional analysis of the *Campylobacter jejuni cj0183* and *cj0588* genes. Curr. Microbiol. 56, 592–596. 10.1007/s00284-008-9130-z 18389311

[B43] Sałamaszynska-Guz A. Taciak B. Kwiatek A. Klimuszko D. (2014). The Cj0588 protein is a *Campylobacter jejuni* RNA methyltransferase. Biochem. Biophys. Res. Commun. 448, 298–302. 10.1016/j.bbrc.2014.04.104 24796671

[B44] Sali A. Blundell T. L. (1993). Comparative protein modelling by satisfaction of spatial restraints. J. Mol. Biol. 234, 779–815. 10.1006/jmbi.1993.1626 8254673

[B45] Sergiev P. V. Golovina A. Prokhorova I. V. Sergeeva O. V. Osterman I. A. Nesterchuk M. V. (2011). Modifications of Ribosomal RNA: from Enzymes to Function Ribosomes: Structure, Function, and Dynamics. Berlin: Springer Verlag.

[B46] Sievers F. Wilm A. Dineen D. Gibson T. J. Karplus K. Li W.. (2011). Fast, scalable generation of high-quality protein multiple sequence alignments using Clustal Omega. Mol. Syst. Biol. 7, 539. 10.1038/msb.2011.75 21988835 PMC3261699

[B47] Stanley R. E. Blaha G. Grodzicki R. L. Strickler M. D. Steitz T. A. (2010). The structures of the anti-tuberculosis antibiotics viomycin and capreomycin bound to the 70S ribosome. Nat. Struct. Mol. Biol. 17, 289–293. 10.1038/nsmb.1755 20154709 PMC2917106

[B48] Svensson S. L. Pryjma M. Gaynor E. C. (2014). Flagella-mediated adhesion and extracellular DNA release contribute to biofilm formation and stress tolerance of *Campylobacter jejuni*. PLoS ONE 9:e106063. 10.1371/journal.pone.0106063 25166748 PMC4148357

[B49] Turonova H. Briandet R. Rodrigues R. Hernould M. Hayek N. Stintzi A.. (2015). Biofilm spatial organization by the emerging pathogen *Campylobacter jejuni*: comparison between NCTC 11168 and 81-176 strains under microaerobic and oxygen-enriched conditions. Front. Microbiol. 6:709. 10.3389/fmicb.2015.00709 26217332 PMC4499754

[B50] Wassenaar T. M. Fry B. N. Van Der Zeijst B. A. (1993). Genetic manipulation of *Campylobacter*: evaluation of natural transformation and electro-transformation. Gene 132, 131–135. 10.1016/0378-1119(93)90525-8 8406035

[B51] Wilson D. N. (2009). The A-Z of bacterial translation inhibitors. Crit. Rev. Biochem. Mol. Biol. 44, 393–433. 10.3109/10409230903307311 19929179

[B52] Witek M. A. Kuiper E. G. Minten E. Crispell E. K. Conn G. L. (2017). A novel motif for S-adenosyl-L-methionine binding by the ribosomal RNA methyltransferase TlyA from *Mycobacterium tuberculosis*. J. Biol. Chem. 292, 1977–1987. 10.1074/jbc.M116.752659 28031456 PMC5290967

[B53] Wren B. W. Stabler R. A. Das S. S. Butcher P. D. Mangan J. A. Clarke J. D.. (1998). Characterization of a haemolysin from *Mycobacterium tuberculosis* with homology to a virulence factor of *Serpulina hyodysenteriae*. Microbiology 144, 1205–1211. 10.1099/00221287-144-5-1205 9611795

[B54] Wrzesinski J. Bakin A. Ofengand J. Lane B. G. (2000). Isolation and properties of *Escherichia coli* 23S-RNA pseudouridine 1911, 1915, 1917 synthase (RluD). IUBMB Life 50, 33–37. 10.1080/15216540050176566 11087118

[B55] Young K. T. Davis L. M. Dirita V. J. (2007). *Campylobacter jejuni*: molecular biology and pathogenesis. Nat. Rev. Microbiol. 5, 665–679. 10.1038/nrmicro1718 17703225

[B56] Yusupov M. M. Yusupova G. Z. Baucom A. Lieberman K. Earnest T. N. Cate J. H.. (2001). Crystal structure of the ribosome at 5.5 Å resolution. Science 292, 883–896. 10.1126/science.1060089 11283358

[B57] Zhang Z. W. Dorrell N. Wren B. W. Farthingt M. J. (2002). *Helicobacter pylori* adherence to gastric epithelial cells: a role for non-adhesin virulence genes. J. Med. Microbiol. 51, 495–502. 10.1099/0022-1317-51-6-495 12018657

