# Supplementary material for: Biofilm Formation and Motility Are Promoted by Cj0588-Directed Methylation of rRNA in Campylobacter jejuni
Source: Front Cell Infect Microbiol. 2018 Jan 18;7:533. doi: 10.3389/fcimb.2017.00533 (PMC5778110; doi:10.3389/fcimb.2017.00533)
Supplement: Supplementary file 1 [file DataSheet1.pdf]

SUPPLEMENTARY MATERIAL

Biofilm formation and motility are promoted by Cj0588-directed rRNA methylation in *Campylobacter jejuni*

Agnieszka Sałamaszyńska-Guz,[1*] Simon Rose,[2] Claus Asger Lykkebo,[2]

Bartłomiej Taciak,[3] Pawel Bacal,[4] Tomasz Uspieński[1] and Stephen Douthwaite[2*]

[1] Division of Microbiology, Department of Pre-Clinical Sciences, Faculty of Veterinary Medicine, Warsaw University of Life Sciences – SGGW, Ciszewskiego 8, 02-786 Warsaw, Poland

[2] Department of Biochemistry and Molecular Biology, University of Southern Denmark, Campusvej 55, DK-5230 Odense M, Denmark.

[3] Division of Physiology, Department of Physiological Sciences, Faculty of Veterinary Medicine, Warsaw University of Life Sciences – SGGW, Nowoursynowska 159, 02-776 Warsaw, Poland

[4] Laboratory of Theory and Applications of Electrodes, Faculty of Chemistry, University of Warsaw, Pasteura 1, 02-093 Warsaw, Poland

Running title: Ribosomal RNA methylation and virulence of *C. jejuni*

Key words: TlyA 2´-*O*-methyltransferase; biofilms; bacterial motility; virulence; capreomycin resistance.

[*]Correspondence to:
Agnieszka Sałamaszyńska-Guz: agnieszka_salamaszynska_guz@sggw.pl
Tel +48 22 593 60 30, Fax: +48 22 593 60 6

Stephen Douthwaite: srd@bmb.sdu.dk; Tel +45 6550 2395

TEXTS FOR SUPPLEMENTARY TABLES

TABLE S1. Bacterial strains and plasmids used in this study.

Km^r: kanamycin resistant; Cm^r: chloramphenicol resistant. Recombinant plasmid structures were tested at each stage by restriction digestion, and plasmids and strains were verified in all cases by PCR sequencing using the primers listed in Table 2.

TABLE S2. Oligonucleotide primers used for cloning and mutagenesis.

The nucleotides underlined indicate restriction recognition sequences that were introduced for cloning purposes: GAATTC, *Eco*RI; and GTCGAC, *Sal*I.

TABLE S1

**Strains**	**Relevant characteristics**	**Source/reference**
C. jejuni 405	Wild type, isolated from poultry	(Rozynek et al., 2008)
C. jejuni 405*Δcj0588*	Cm^r, *cj0588* deletion mutant	This study
C. jejuni 405*Δcj0588* + pMW0588	Cm^r, Km^r, *cj0588* deletion mutant complemented with pMW0588	This study
C. jejuni 405*Δcj0588* + pMW0588-K80A	Cm^r, Km^r, *cj0588* deletion mutant complemented with pMW0588-K80A	This study
C. jejuni 405*Δcj0588* + pMW0588-D162A	Cm^r, Km^r, *cj0588* deletion mutant complemented with pMW0588-D162A	This study
C. jejuni 405*Δcj0588* + pMW0588-K188A	Cm^r, Km^r, *cj0588* deletion mutant complemented with pMW0588-K188A	This study
C. jejuni 81-176	Wild type	(Korlath et al., 1985)
C. jejuni 81-176*Δcj0588*	Cm^r, *cj0588* deletion mutant	This study
C. jejuni 81-176*Δcj0588::0588*	Cm^r, Km^r, *cj0588* deletion mutant complemented with wild-type *cj0588*	This study
C. jejuni 81-176*Δcj0588::0588*-K80A	Cm^r, Km^r, *cj0588* deletion mutant complemented with the *cj0588* with the K80A mutation	This study
C. jejuni 81-176*Δcj0588::0588*-D162A	Cm^r, Km^r, *cj0588* deletion mutant complemented with *cj0588* with the D162A mutation	This study
C. jejuni 81-176*Δcj0588::0588*-K188A	Cm^r, Km^r *cj0588* deletion mutant complemented with *cj0588* with the K188A mutation	This study
E. coli BL21(DE3)	F$^-$ *ompT hsdS*$_B$ (r_B^- m_B^-) *gal dcm* (DE3)	Novagen
E. coli BL21(DE3) + pET0588	*E. coli* BL21(DE3) carrying pET expressing the wild-type *cj0588* gene	(Salamaszynska-Guz and Klimuszko, 2008)
E. coli K80A	*E. coli* BL21(DE3) carrying pET expressing *cj0588* where lysine-80 is substituted with alanine	This study
E. coli D162A	*E. coli* BL21(DE3) carrying pET expressing *cj0588* where aspartate-162 is substituted with alanine	This study
E. coli K188A	*E. coli* BL21(DE3) carrying pET expressing *cj0588* where lysine-188 is substituted with alanine	This study
**Plasmids**		
p0183	pMW10 (*aphA*, Km^r) shuttle vector with cloned 450-bp region upstream of *cj0183*	(Salamasznska-Guz et al., 2013)
pMW0588	p0183 with *cj0588* under *C. jejuni* promoter control	This study
pMW0588-K80A, etc.	pMW0588 with the designated substitution	This study
pET28a	T7 promoter expression vector, Km^r	Novagen
pET0588	pET28a with *cj0588* gene	(Salamaszynska-Guz and Klimuszko, 2008)

TABLE S2

Primer	Primer sequence (5´– 3´) and application
588E	GAATTC**ATG**AGATTTGATTTTTTTGTTTCA
588S	GTCGACATTTTTGATATAGTAGTAAA
C5F	TTCTTGGCGATTTTTGGACTT: Amplification of *cj0588* by PCR
C5R	AAGCCCCATTCTCATCTAAACA: Amplification of *cj0588* by PCR
catF	GAATTCAGCTGCTCGGCGGTGTTCCTTTCCAAG: *cat* gene, plasmid/null-mutant screening
catR	GAATTCAGCTGCGCCCTTTAGTTCCTAAAGGGT: *cat* gene, plasmid/null-mutant screening
C2	ATACCATCAAAACAACCTCTGGCTA
K80AF	GCCAAAGCTGCTCTTGAAACATAAATTTCACTTAAAAG
K80AR	ATTAAAAAAATTTTTAGAAGAAAATGATATTGAAATAAAACATAAAAATTGTCTTGATATAGG
D162AF	GTAAATTAATAAGAGAAATAAAACTCACAGCACAAGTAACAAGTTCAAATTTTTCTTCA
D162AR	TGAAGAAAAATTTGAACTTGTTACTTGTGCTGTGAGTTTTATTTCTCTTATTAATTTAC
K188AF	GCAAAAAGTAAAATAATTTCCTTTAAAGCTAAATTATCAATATAATAAAGTAAATTAATAAG
K188AR	ACCTCAGTTTGAAGTGGGAAAAAATATCAAAAGAGATAAAAAAG
SR197	GTGAAATCAACTCCCATGG<br>Primer extension. Complementary to *C. jejuni* 16S rRNA nucleotides 1411-1429
SR198	GAATTTCGCTACCTTAGG<br>Primer extension. Complementary to *C. jejuni* 23S rRNA nucleotides 1924-1941
SR199	GAGTGAAATCAACTCCCATGGTGTGACGGGCGGTGAGTACAAGACCCG<br>MS analysis; hybridization to *C. jejuni* 16S rRNA nucleotides 1384-1431
SJ20	CGACAAGGAATTTCGCTACCTTAGGACCGTTATAGTTACGGCCGCCGTTTACCGGGGCTT<br>MS analysis; hybridization to *C. jejuni* 23S rRNA nucleotides 1889-1948

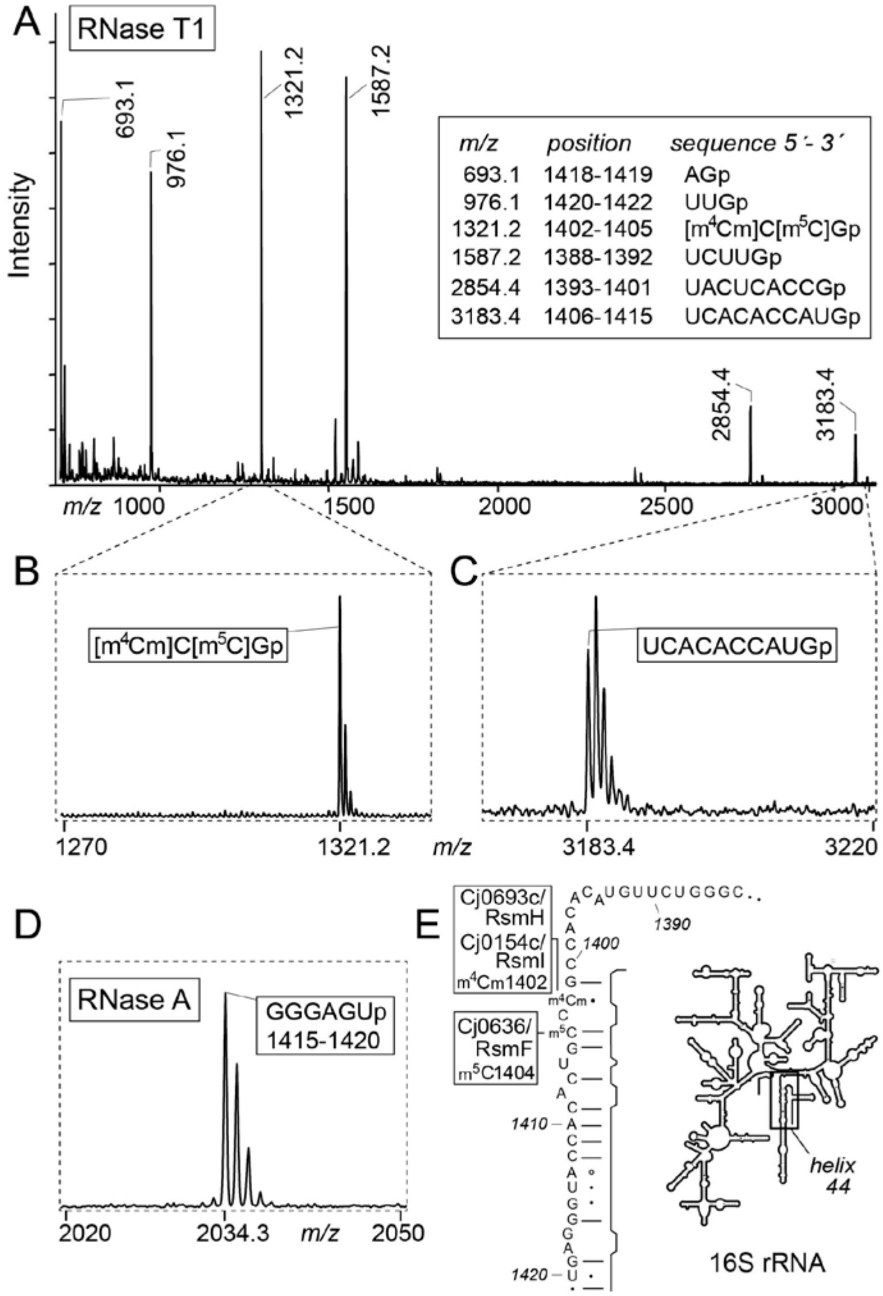

FIGURE S1 MS analyses of *C. jejuni* 16S rRNA. **(A)** Spectrum of RNase T1 fragments derived the 48-nucleotide sequence encompassing C1409 in 16S rRNA from the *C.jejuni* 405 wild-type strain. The mass/charges (*m/z*) of these fragments remained unchanged in all the mutant and complemented strains. **(B)** Enlargement of the spectral region around the CCCGp fragment at *m/z* 1321.2, which contains three methyl groups (two on nucleotide C1402 and one on C1404). **(C)** The UCACACCAUGp fragment from 1406-1415 contains nucleotide C1409 and formed a peak at *m/z* 3183.4 showing that this sequence is unmodified. **(D)** The RNase A fragment of the neighboring sequence from 1415-1420 showing that there were no other modifications in this region. **(E)** Helix 44 of the 16S rRNA (boxed in the 16S schematic) showing part of the sequence that was analyzed by MS with the sites of modification and the *C. jejuni* enzymes that presumably catalyze the reactions.

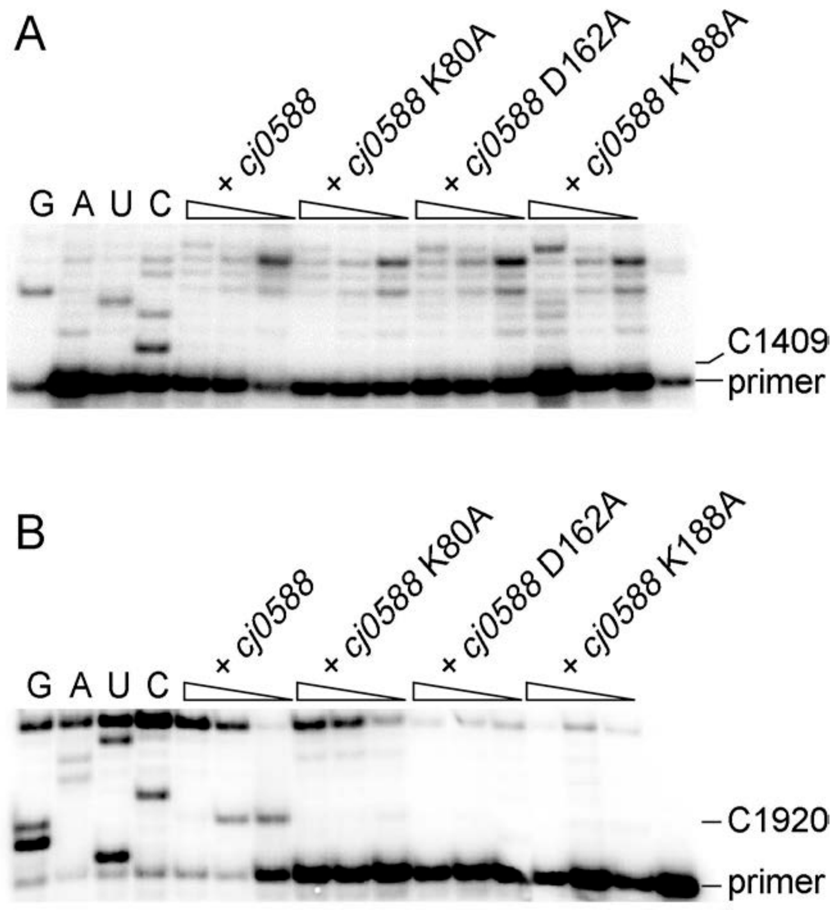

FIGURE S2 Gel autoradiograms of primer extensions on rRNA from *C. jejuni* strains containing Cj0588 variants. **(A)** Extension on 16S rRNAs, and **(B)** 23S rRNAs extracted from the *C. jejuni* 405 null-strain complemented with pMW plasmid-encoded wild-type *cj0588* (+ *cj0588*), or the K80A, D162A or K188A versions of *cj0588*. Decreasing dGTP concentrations (indicated by wedges) intensifies reverse transcription pausing at cytidines with 2´-*O*-methylation (Maden et al., 1995). Methylation occurred only at 23S rRNA nucleotide C1920 in the strain complemented with wild-type Cj0588. Lanes G, A, U and C are dideoxy-sequencing reactions on unmodified *C. jejuni* rRNAs.

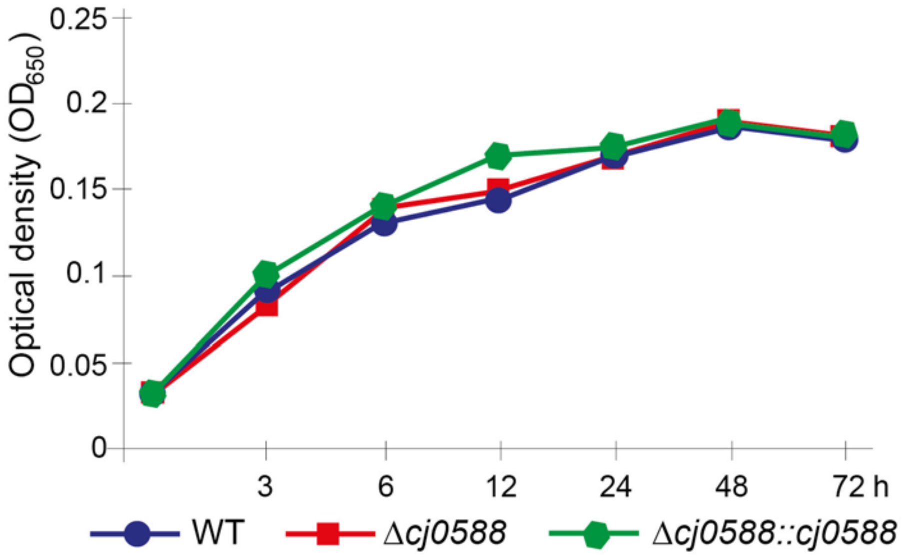

FIGURE S3 Growth of *C. jejuni* 81167 strains over 72 h (nonlinear time scale) under microaerobic conditions at 37°C in Mueller-Hinton liquid medium. WT is the wild-type 81167 strain; Δ*cj588* is the *cj0588*-null mutant; and Δ*cj588::cj588* is the null-mutant complemented by insertion of an active copy of the *cj0588* gene in the chromosome. All growth points are the means of OD_{650} values from three independent cultures. Under these conditions, there was no significant difference in the growth rate of any of the strains.

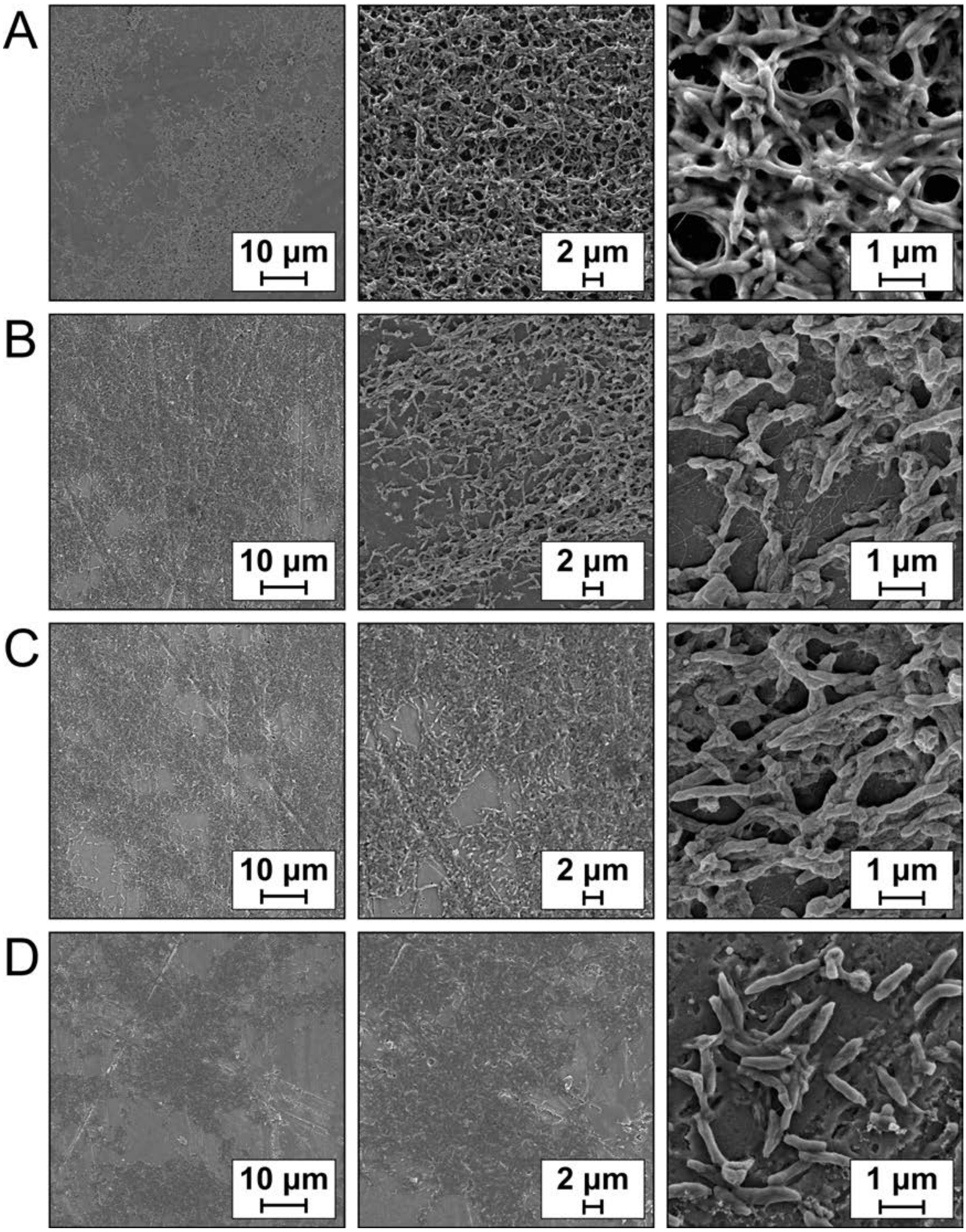

FIGURE S4 Biofilm produced by *C. jejuni* 81-176 on cover glass after 48 h under microaerobic conditions at 37°C visualized by Field Emission Scanning Electron Microscopy. The *C. jejuni* 81-176 null mutant complemented by insertion of **(A)** an active copy of the *cj0588* gene in the chromosome (Δ*cj0588::cj0588*, as in Figure 7C), or **(B)** with the K80A-mutant copy of *cj0588*, or **(C)** the D162A-mutant copy of *cj0588*, or **(D)** the K188A-mutant copy of *cj0588*. Triplicate cultures of all strains were viewed and representative micrographs are shown.

References

Korlath, J. A., Osterholm, M. T., Judy, L. A., Forfang, J. C. & Robinson, R. A. 1985. A point-source outbreak of campylobacteriosis associated with consumption of raw milk. *J Infect Dis,* 152**,** 592-6.

Maden, B. E. H., Corbett, M. E., Heeney, P. A., Pugh, K. & Ajuh, P. M. 1995. Classical and novel approaches to the detection and localization of the numerous modified nucleotides in eukaryotic ribosomal RNA. *Biochimie,* 77**,** 22-29.

Rozynek, E., Dzierzanowska-Fangrat, K., Korsak, D., Konieczny, P., Wardak, S., Szych, J., Jarosz, M. & Dzierzanowska, D. 2008. Comparison of antimicrobial resistance of *Campylobacter jejuni* and *Campylobacter coli* isolated from humans and chicken carcasses in Poland. *J Food Prot,* 71**,** 602-7.

Salamasznska-Guz, A., Grodzik, M. & Klimuszko, D. 2013. Mutational analysis of *cj0183 Campylobacter jejuni* promoter. *Curr Microbiol,* 67**,** 696-702. doi 10.1007/s00284-013-0420-8.

Salamaszynska-Guz, A. & Klimuszko, D. 2008. Functional analysis of the *Campylobacter jejuni cj0183* and *cj0588* genes. *Curr Microbiol,* 56**,** 592-6. doi 10.1007/s00284-008-9130-z.
